# Plasmalogen-Based Liquid Crystalline Multiphase Structures Involving Docosapentaenoyl Derivatives Inspired by Biological Cubic Membranes

**DOI:** 10.3389/fcell.2021.617984

**Published:** 2021-02-11

**Authors:** Angelina Angelova, Borislav Angelov, Markus Drechsler, Thomas Bizien, Yulia E. Gorshkova, Yuru Deng

**Affiliations:** ^1^Institut Galien Paris-Saclay UMR8612, Université Paris-Saclay, CNRS, Châtenay-Malabry, France; ^2^Institute of Physics, ELI Beamlines, Academy of Sciences of the Czech Republic, Prague, Czech; ^3^Keylab “Electron and Optical Microscopy”, Bavarian Polymer Institute, University of Bayreuth, Bayreuth, Germany; ^4^Synchrotron SOLEIL, L’Orme des Merisiers, Saint-Aubin, France; ^5^Frank Laboratory of Neutron Physics, Joint Institute for Nuclear Research, Dubna, Russia; ^6^Wenzhou Institute, University of Chinese Academy of Sciences, Wenzhou, China

**Keywords:** docosapentaenoyl phospholipids, lipid cubic phase, inverted hexagonal phase, plasmalogen-loaded cubosomes, hexosomes, SAXS, cryo-TEM

## Abstract

Structural properties of plasmenyl-glycerophospholipids (plasmalogens) have been scarcely studied for plasmalogens with long polyunsaturated fatty acid (PUFA) chains, despite of their significance for the organization and functions of the cellular membranes. Elaboration of supramolecular assemblies involving PUFA-chain plasmalogens in nanostructured mixtures with lyotropic lipids may accelerate the development of nanomedicines for certain severe pathologies (e.g., peroxisomal disorders, cardiometabolic impairments, and neurodegenerative Alzheimer’s and Parkinson’s diseases). Here, we investigate the spontaneous self-assembly of bioinspired, custom-produced docosapentaenoyl (DPA) plasmenyl (ether) and ester phospholipids in aqueous environment (pH 7) by synchrotron small-angle X-ray scattering (SAXS) and cryogenic transmission electron microscopy (cryo-TEM). A coexistence of a liquid crystalline primitive cubic *Im3m* phase and an inverted hexagonal (H_II_) phase is observed for the DPA-ethanolamine plasmalogen (C16:1p-22:5n6 PE) derivative. A double-diamond cubic *Pn3m* phase is formed in mixed assemblies of the phosphoethanolamine plasmalogen (C16:1p-22:5n6 PE) and monoolein (MO), whereas a coexistence of cubic and lamellar liquid crystalline phases is established for the DPA-plasmenyl phosphocholine (C16:1p-22:5n6 PC)/MO mixture at ambient temperature. The DPA-diacyl phosphoinositol (22:5n6-22:5n6 PI) ester lipid displays a propensity for a lamellar phase formation. Double membrane vesicles and multilamellar onion topologies with inhomogeneous distribution of interfacial curvature are formed upon incorporation of the phosphoethanolamine plasmalogen (C16:1p-22:5n6 PE) into dioleoylphosphocholine (DOPC) bilayers. Nanoparticulate formulations of plasmalogen-loaded cubosomes, hexosomes, and various multiphase cubosome- and hexosome-derived architectures and mixed type nano-objects (e.g., oil droplet-embedding vesicles or core–shell particles with soft corona) are produced with PUFA-chain phospholipids and lipophilic antioxidant-containing membrane compositions that are characterized by synchrotron SAXS and cryo-TEM imaging. The obtained multiphase nanostructures reflect the changes in the membrane curvature induced by the inclusion of DPA-based PE and PC plasmalogens, as well as DPA-PI ester derivative, and open new opportunities for exploration of these bioinspired nanoassemblies.

## Introduction

Plasmenyl-phospholipids (plasmalogens) are a class of ether-type (1-alkyl-1′-enyl, 2-acyl) glycerophospholipids with essential functions for the living cells in the brain, retina, heart, lung, skeletal muscles, and testis (Gross, 1985; Creuwels et al., 1997; Batenburg and Haagsman, 1998; Snyder, 1999; Goldfine, 2010; Wallner and Schmitz, 2011; Saab et al., 2014; Dean and Lodhi, 2018; Jiménez-Rojo and Riezman, 2019; Paul et al., 2019). They are recognized as crucial for the human health considering that plasmalogens deficiency is associated with the disease progress in various pathologies (Han et al., 2001; Munn et al., 2003; Brites et al., 2004; Braverman and Moser, 2012; Brosche et al., 2013; Sutter et al., 2015; Jang et al., 2017; Messias et al., 2018). Decreased ethanolamine plasmalogen levels have been established with the progression of Alzheimer’s (AD) and Parkinson’s (PD) diseases, psychiatric disorders, and also in neuromuscular impairments, glaucoma, coronary artery disease, and acute myocardial infarction (Farooqui et al., 1997; Dragonas et al., 2009; Brodde et al., 2012; Sutter et al., 2016; Dorninger et al., 2017a, b, 2019; Paul et al., 2019; Su et al., 2019). The plasmalogen lipids modulate the membrane fluidity and dynamics, influence the membrane protein organization, and provide reservoirs of secondary messengers as well as precursors of inflammatory mediators (Bogdanov et al., 1999; Thai et al., 2001; Mannock et al., 2010; Koivuniemi, 2017; Jenkins et al., 2018; Pohl and Jovanovic, 2019; Fontaine et al., 2020). The vinyl ether moiety at the sn-1 position of glycerol backbone is significant for the antioxidant (scavenger) properties of plasmalogens and the protection of neuronal, cardiac, and muscle cells from oxidative stress (Reiss et al., 1997; Zoeller et al., 1999; Leßig and Fuchs, 2009; Wallner and Schmitz, 2011; Yamashita et al., 2015; Sibomana et al., 2019).

**FIGURE 1:**
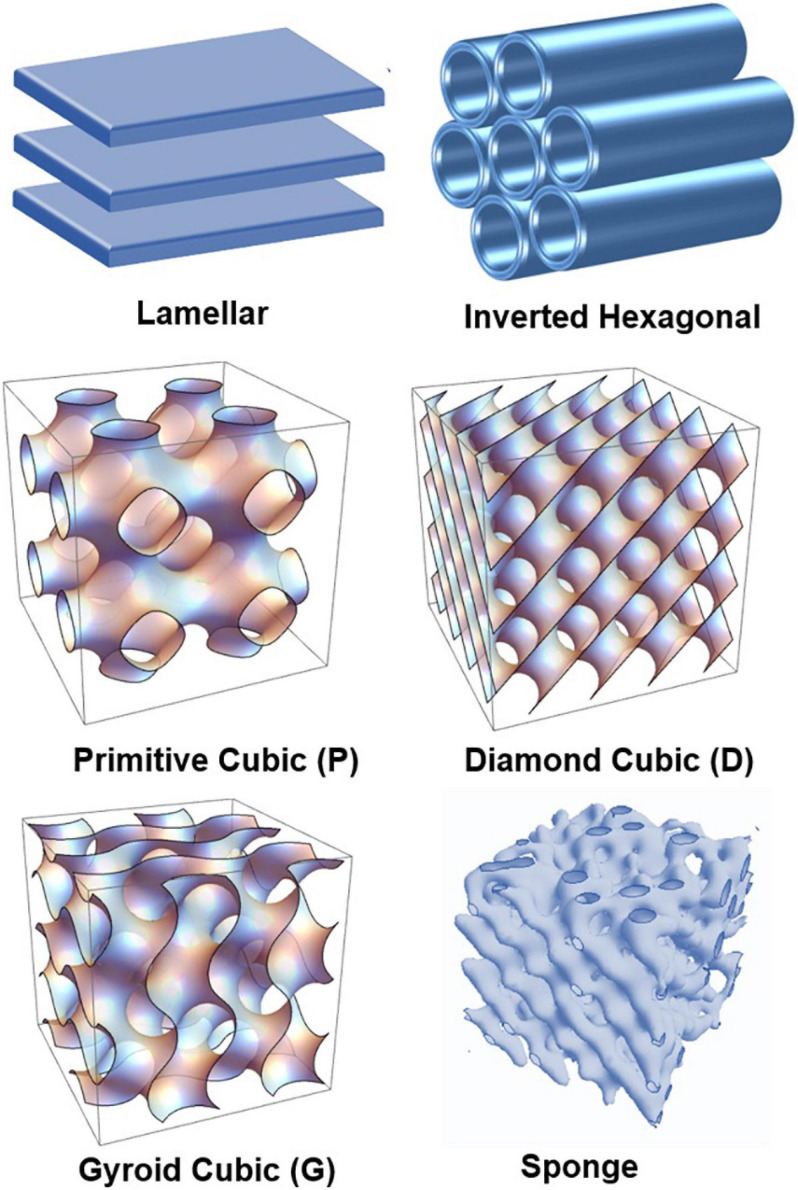
Schematic presentation of lamellar and non-lamellar liquid crystalline phase structures resulting from the structural polymorphism of lyotropic lipid/water mixtures: lamellar bilayer, inverted hexagonal (H_II_), primitive cubic (P), bicontinuous double-diamond cubic (D), bicontinuous gyroid cubic (G), and sponge phases.

In this work, we aim at deeper understanding of the structural polymorphism of hydrated polyunsaturated fatty acid (PUFA) plasmalogens and esters and their capacity to form self-assembled nanoscale structures as pure lyotropic lipids or in mixtures with other amphiphilies. Figure 1 presents the major polymorphic states of hydrated lyotropic lipids (Mariani et al., 1988; Seddon and Templer, 1995). The arrangement of lipids and amphiphiles in supramolecular assemblies is governed by intermolecular hydrophobic and electrostatic interactions, temperature, degree of hydration, and the geometry of the molecules (Israelachvili et al., 1976; Cullis and de Kruijff, 1979; Cui et al., 2007; Shaharabani et al., 2016).

The non-lamellar phase-forming tendencies of some ether lipid types have been evaluated in an isolated state based on measurements of their lamellar-gel to lamellar-fluid chain-melting characteristics and also of lamellar-to-non-lamellar inverted hexagonal (H_II_) phase transition temperatures (Goldfine et al., 1981; Malthaner et al., 1987). Such parameters have been determined for ethanolamine plasmalogen derivatives extracted from egg, beef heart, or *Clostridium butyricum* (Goldfine et al., 1987a) using differential scanning calorimetry (DSC) and nuclear magnetic resonance (^31*P*^NMR) spectroscopic studies of lipid membrane systems (Boggs et al., 1981; Lohner et al., 1984; Malthaner et al., 1987). It has been intriguing to establish whether the non-lamellar phase behavior of plasmalogen lipids is a result of the location of carbon–carbon double bond (Goldfine et al., 1981, 1987a). The presence of 1′-carbon–carbon double bond at sn-1 position of the glycerol backbone of ethanolamine plasmalogens has been expected to influence lipid polymorphism through the degree of polarity of polar–apolar interfaces of lipid membranes (Goldfine et al., 1987b; Rog and Koivuniemi, 2016).

High-resolution structural data have been required to better understand the formation of non-lamellar liquid crystalline phases by plasmenyl phospholipids and the relationship between molecular structure and phase behavior (Figure 1). Such studies have been initiated in the past for plasmalogens with C16:0, C18:0, and C18:1 aliphatic moieties (Lohner et al., 1984, 1991). The performed small-angle X-ray scattering (SAXS) investigations have indicated that the induction of non-lamellar phases, in particular of an inverted hexagonal (H_II_) phase, is promoted by the increase in the hydrocarbon chain length (e.g., from C16 to C18) as well as by the increase in the degree of chain unsaturation (e.g., from di-C18:1 to di-C18:2; Lohner, 1996). The hydration of the headgroups and their interaction with the solvent has also been an important determinant for the physicochemical differences, which have been observed in the phase behaviors of the studied lipid types (Lohner et al., 1984). To our knowledge, structural SAXS data are not available yet for plasmalogens with PUFA chains.

Ultrastructural studies of subcellular organelles by transmission electron microscopy (TEM) have indicated the role of plasmalogens in the induction of non-lamellar cubic membrane structures under starvation stress conditions (Chong and Deng, 2012; Deng et al., 2017). From a structural viewpoint, docosapentaenoic acid (DPA) has been identified as the critical PUFA determinant for the cubic lipid membrane formation in amoeba *Chaos* mitochondria (Deng et al., 2009; Almsherqi et al., 2010). It has been emphasized that plasmalogens are structural membrane components that act also as antioxidants in the *Chaos* cells. The survival mechanism (from the applied cellular stress) has been explained by the formation of nanoperiodic arrangements of biological cubic membranes during cellular stress response to unfavorable environmental cues (Deng and Almsherqi, 2015). The presence of cubic membranes has been associated with improved cell survival during long-term starvation (Deng et al., 2009). In addition, it has been reported that the inverted hexagonal (H_II_) phase of plasmenyl-phosphoethanolamines promotes the membrane fusion (Boggs et al., 1981; Han and Gross, 1990; Glaser and Gross, 1994).

The lack of effective treatments of neurodegenerative diseases is an urgent challenge at present. Nanoparticles and nanoassemblies may offer innovative combinations of multiple bioactive molecules to target the various mechanisms of multiple neurodegenerative diseases (Angelova et al., 2011, 2019b; Fonseca-Santos et al., 2015; Angelova and Angelov, 2017; Harilal et al., 2019). In recent works, we have exploited self-assembly-based nanotechnologies for combination delivery of a key neurotrophic protein [brain-derived neurotrophic factor (BDNF)], curcumin, and omega-3 polyunsaturated fatty acids (ω-3 PUFAs) in order to trigger and promote neuro-repair (Guerzoni et al., 2017; Angelova et al., 2018; Rakotoarisoa et al., 2019). Biomedical studies have emphasized that AD patients lose up to 60% of plasmalogens from the brain cell membranes (Farooqui et al., 1997; Su et al., 2019). There is rising evidence that phospholipids of the plasmalogen-phosphatidylethanolamine type may provide a novel therapeutic approach to impact the neuronal functions in AD and PD pathologies (Hossain et al., 2013; Yamashita et al., 2015; Che et al., 2018, 2020; Mawatari et al., 2020). Results from recent clinical trials have shown that PUFA-chain ethanolamine plasmalogens can be 100-fold more powerful in stimulating neuro-repair as compared to the conventional ω-3 PUFA species (Fujino et al., 2017; Fujino et al., 2018). Biological assays also revealed that the suppression of the neuronal cell apoptosis by plasmalogens is more efficient for lipids with longer PUFA chains such as C18:0/p-22:6-PE or C18:0/p-22:6-PC (Yamashita et al., 2015; Che et al., 2020). Hence, further investigations of PUFA-chain phospholipid phase behavior, miscibility in lipid bilayers, effects on membrane curvature, membrane fusion, formation of transient, or permanent nanoperiodic arrangements, compartmentalization (Muallem et al., 2017; Bharadwaj et al., 2018; Casares et al., 2019), and nanostructure formation are needed in order to define prospective nanomedicine-based therapeutic strategies.

In this context, we report here a structural study of novel custom-synthesized plasmenyl (ether) and ester phospholipids with long PUFA (22:5n6) chains. They were designed by bioinspiration from the main constituents of the biological cubic membranes identified in mitochondria of starved amoeba *Chaos* cells (Deng et al., 2009; Almsherqi et al., 2010; Deng and Almsherqi, 2015; Deng et al., 2017). The choice of the docosapentaenoyl (DPA, 22:5n6) PUFA chains was determined by the lipidomic (gas liquid chromatography and mass spectrometry) analysis of membranous compositions extracted from amoeba *Chaos* mitochondria (Deng et al., 2009; Almsherqi et al., 2010).

**FIGURE 2:**
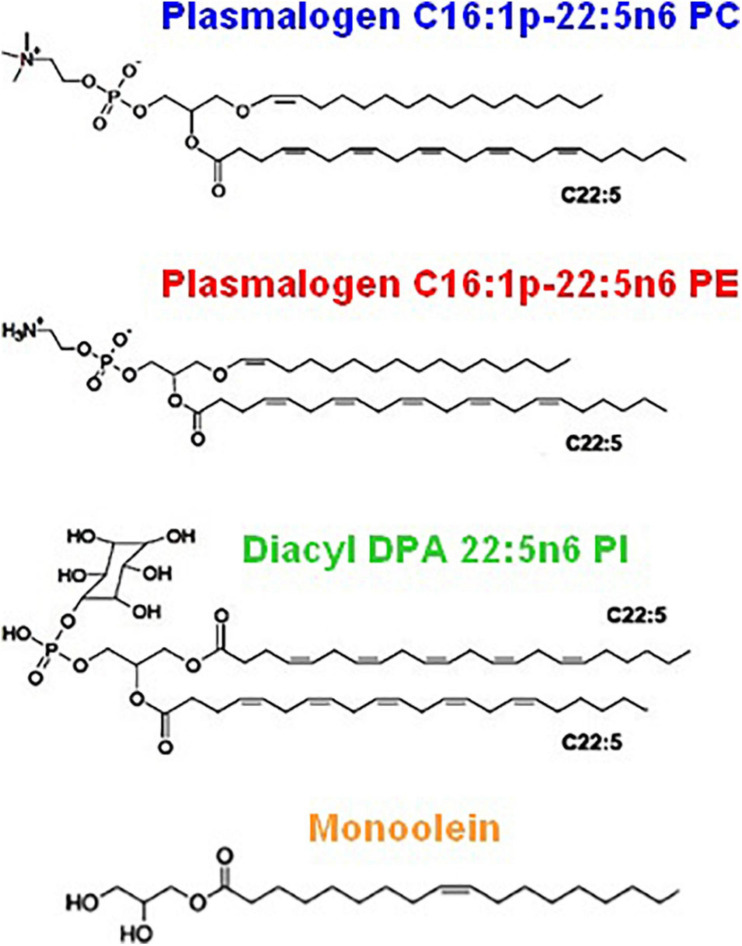
Chemical structures of custom-produced polyunsaturated fatty acid (PUFA)-ether or ester type glycerophospholipids with long tails comprised of docosapentaenoyl (DPA) chains. Plasmalogens (1-alkyl-1′-enyl,2-acyl ether phospholipids) are obtained with phosphocholine and phosphoethanolamine headgroups, namely, 1-O-1′-(Z)-hexadecenyl-2-(4Z,7Z,10Z,13Z,16Z-docosapentaenoyl)-sn-glycero-3-phosphocholine, C16:1p-22:5n6 PC (top), and 1-O-1′-(Z)-hexadecenyl-2-(4Z,7Z,10Z,13Z,16Z-docosapentaenoyl)-sn-glycero-3-phosphoethanolamine, C16:1p-22:5n6 PE (middle). As a diacyl ester lipid, 1,2-bis(4Z,7Z,10Z,13Z,16Z-docosapentaenoyl)-sn-glycero-3- phosphoinositol, 22:5n6 PI, is enriched in docosapentaenoyl (DPA) moieties in order to increase the hydrophobic lipid volume with regard to the bulky phosphoinositol (PI) polar headgroup. Monoolein (bottom) is a single-chain monoglyceride used as a colipid in the studied mixed nanoassemblies.

Figure 2 shows the chemical structures of the custom-synthesized PUFA-chain phospholipid compounds, namely, plasmenyl phosphoethanolamine (C16:1p-22:5n6 PE), plasmenyl phosphocholine (C16:1p-22:5n6 PC), and DPA-diacyl phosphoinositol (22:5n6-22:5n6 PI) ester. The formation of lyotropic liquid crystalline phases by these synthetic phospholipids and their mixtures with a non-lamellar lipid monoolein (MO) or a lamellar phospholipid DOPC, serving as matrix colipids, was studied by synchrotron SAXS at selected compositions. Stable dispersions of nanostructured particles, involving PUFA-chain phospholipids and natural small molecule antioxidants (vitamin E and coenzyme Q10), were produced. The effects of PUFA-chain phospholipids (plasmalogens or esters) on the membrane curvature were exploited in order to create liquid crystalline nanoparticles with different topologies and inner organizations, which were evidenced by SAXS and cryo-TEM imaging. Then, the significance of the particle topologies, crystalline organizations, and compartmentalization for prospective biomedical applications is discussed.

## Results

### Self-Assembled Bulk Liquid Crystalline Lipid/Water Phases of Docosapentaenoyl-Plasmalogens and DPA-Phospholipid Constituents Chosen by Bioinspiration From Biological Cubic Membranes

The structural polymorphism of synthetic PUFA-phospholipid/water phases resulting from the rationally chosen lipid chemical structures is characterized here by synchrotron SAXS. The following features are considered: (i) structural role of the hydrocarbon chains through a study of synthetic plasmalogen derivatives with long polyunsaturated DPA (C22:5n6) chains and (ii) structural role of the polar headgroups through a study of DPA-glycerophospholipids with PE, PC, and PI headgroups. The SAXS investigations were performed under neutral pH condition taking into account that the alkenyl (vinyl ether) bond in the plasmalogen molecules is susceptible to hydrolysis at strong acidic pH.

It is of great interest to find out whether the PUFA-chain plasmalogens and esters involving docosapentaenoyl (DPA, C22:5n6) tails (Figure 2) may generate non-lamellar liquid crystalline phases (Figure 1) upon self-assembly in a pure state in aqueous environment as well as whether they may induce non-lamellar bicontinuous cubic phase formation upon mixing with membrane lipids in synthetic self-assembled systems. These findings should add new knowledge about (i) the membrane curvature-modifying capacity of the designed DPA-phospholipid derivatives and (ii) the usefulness of nanocrystalline arrangements for the development of non-lamellar nanoparticles of bioactive lipids.

#### Non-lamellar Cubic and Inverted Hexagonal Phases Formed by Hydrated DPA-Plasmalogen Phosphoethanolamine

**FIGURE 3:**
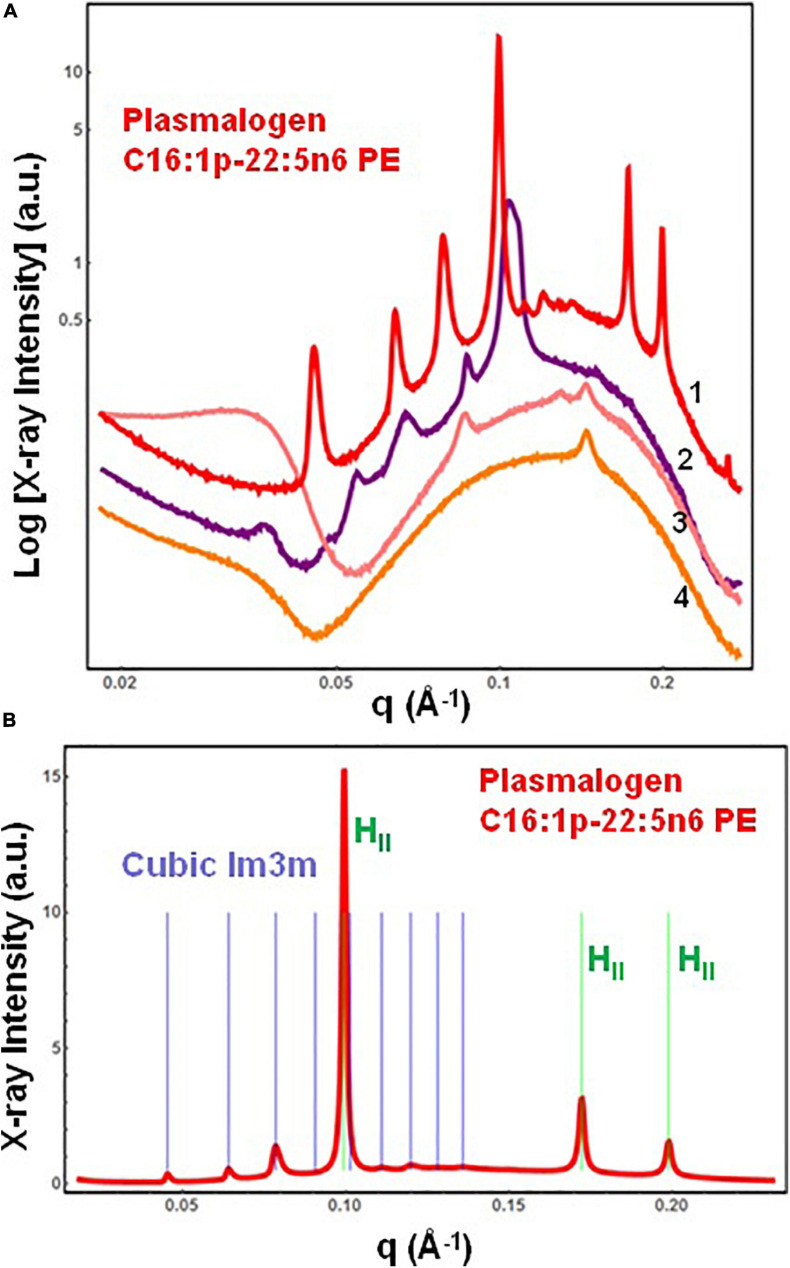
**(A)** Synchrotron small-angle X-ray scattering (SAXS) patterns of a hydrated bulk-phase plasmenyl-phospholipid [DPA-ethanolamine plasmalogen (C16:1p-22:5n6 PE)] at a lipid/water ratio of 50/50 (wt%/wt%) and aqueous phosphate buffer environment (1.10^− 2^ M) of pH 7 with added 2,6-di-tert-butyl-4-methylphenol (BHT). Pattern (1) corresponds to a freshly prepared plasmalogen/water sample examined by synchrotron SAXS. Plots (2)–(4) display the progressive vanishing of the cubic phase peaks upon multiple SAXS recordings with the same sample. **(B)** Indexing of the Bragg diffraction peaks resolved in the SAXS pattern (1) from **(A)**. In a linear scale, the two sets of Bragg peaks index: (i) a primitive cubic phase of the *Im3m* space group of symmetry [purple bars corresponding to the (110), (200), (211), (220), (310), (222), (321), (400), and (411) cubic lattice reflections for peak positions spaced in the ratio √2:√4:√6:√8:√10:√12:√14:√16:√18] and (ii) an inverted hexagonal (H_II_) phase [green bars corresponding to the (10), (11), and (20) reflections at peak positions spaced in the ratio 1:√3:√4]. Temperature is 22°C.

The SAXS patterns of the DPA-phosphoethanolamine plasmalogen (C16:1p-22:5n6 PE) in a hydrated bulk state are presented in Figure 3A. The investigated hydration level is determined by a lipid/water ratio of 50/50 (wt/wt). The positions of Bragg diffraction peaks detected in the SAXS pattern 1 (red plot) are indexed in Figure 3B. A coexistence of a liquid crystalline primitive cubic *Im3m* phase with an inverted hexagonal (H_II_) phase is identified at a temperature of 22°C for the freshly prepared bulk-phase DPA-ethanolamine plasmalogen (C16:1p-22:5n6 PE)/water system (Figure 3A, plot 1). The first Bragg peak of the primitive cubic *Im3m* phase is resolved at *q* = 0.0453 Å^–1^, while the first Bragg peak of H_II_ phase is recorded at *q* = 0.0994 Å^–1^ (Figure 3B). The *Im3m* cubic lattice parameter (estimated from the sequence of Braggs peaks spaced in the ratio √2:√4:√6:√8:√10:√12:√14:√16:√18) is a_*Q(Im*__3__*m)*_ = 19.6 nm. The H_II_-phase is characterized by a lattice parameter of a_H_ = 7.23 nm. A closer examination of the SAXS plot in Figure 3B reveals an overlap of a Bragg diffraction peak of the primitive cubic *Im3m* phase with the first Bragg peak of the inverted hexagonal (H_II_) phase at *q* = 0.0994 Å^–1^. This coexistence characterizes the polymorphism of the liquid crystalline DPA-plasmenyl-PE/water system, which was studied at room temperature. The fact that the PE-plasmalogen does not form a unique stable non-lamellar mesophase suggests that the flexible long polyunsaturated DPA chains may impart a propensity for the formation of domain structures of this lipid in biological membranes.

The SAXS plots (2)–(4) in Figure 3A demonstrate the radiation-sensitive behavior of the hydrated plasmalogen (C16:1p-22:5n6 PE) assembly. The synthetic lipid membranes undergo a cubic-to-hexagonal phase transition, which reaches a weakly ordered lipid state upon repeated exposure to the X-ray beam. The Bragg peaks of the primitive *Im3m* cubic phase progressively vanish owing to the radiation-induced interconversion. The peaks of the inverted hexagonal (H_II_) phase are detectable in the SAXS plots (2)–(4) in Figure 3A and thus represent a longer-lived, more stable non-lamellar structure.

#### Cubic Phases and Structural Intermediates in Two-Component Bulk Phases of Docosapentaenoyl-Phospholipids and Monoolein

**FIGURE 4:**
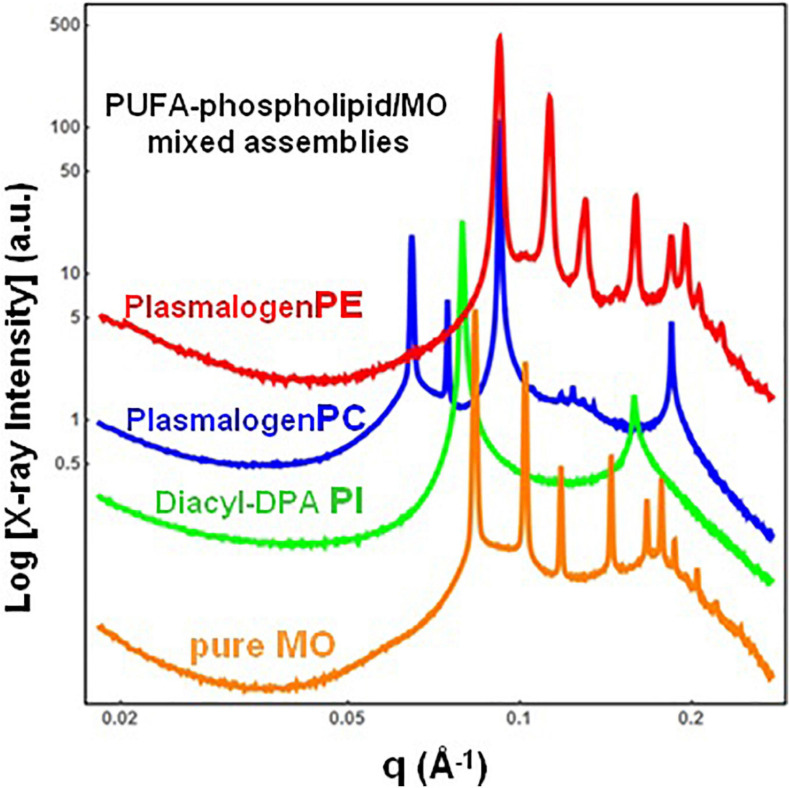
Synchrotron small-angle X-ray scattering (SAXS) patterns of bulk-phase mixtures of monoolein (MO) with incorporated 15 mol.% docosapentaenoyl (DPA, 22:5n6)-modified plasmalogen-phosphoethanolamine C16:1p-22:5n6 PE (red plot), plasmalogen phosphocholine C16:1p-22:5n6 PC (blue plot), or DPA-diacyl phosphoinositol 22:5n6-22:5n6 PI ester lipid (green plot). The red, blue, and green plots correspond to 15/85 (mol/mol) ratio between the polyunsaturated fatty acid (PUFA) phospholipids and monoolein (MO). The orange plot presents the SAXS pattern for the pure MO/buffer system. The lipid/water ratio is 40/60 (wt/wt). Aqueous phase: 1.10^− 2^ M phosphate buffer containing 2,6-di-tert-butyl-4-methylphenol (BHT). Temperature is 22°C.

The single-component DPA-plasmalogen did not form a stable unique bicontinuous cubic phase at room temperature (Figure 3). Taking into account the instability on storage of the cubic *Im3m* liquid crystalline phase of the hydrated DPA-plasmalogen and the observed radiation-induced interconversion of the single-lipid system (Figure 3), mixed lipid phases were subsequently formulated using a colipid. The lyotropic single-chain lipid monoolein (MO; Figure 2) was added at a selected molar ratio to the studied double-chain ether or ester phospholipids involving polyunsaturated (22:5n6) tails. The impact of 15 mol.% DPA-phospholipid incorporation on the structural organization of self-assembled bulk two-component lipid/water phases was evidenced by SAXS. Under the investigated conditions, hydrated MO forms a bicontinuous cubic phase of the double-diamond *Pn3m* space group of symmetry (Figure 4, orange plot). The recorded first Bragg peak is centered at *q* = 0.083 Å^–1^. It belongs to a set of Bragg peaks, spaced in the ratio √2:√3:√4:√6:√8:√9:√10:√11:√12:√14 …, which determines a cubic lattice parameter a_Q(Pn__3__*m*__)_ = 10.8 nm.

The red plot in Figure 4 shows the SAXS pattern of a hydrated MO assembly incorporating 15 mol.% DPA-ethanolamine plasmalogen (C16:1p-22:5n6 PE). The PE-plasmalogen, containing a single polyunsaturated (22:5n6) fatty acid chain (Figure 2), preserves the non-lamellar organization of the host lipid/water system. The sequence of Bragg diffraction peaks, spaced in the ratio √2:√3:√4:√6:√8:√9:√10:√11:√12 … (with a first maximum centered at *q* = 0.092 Å^–1^), defines a distinct bicontinuous cubic phase structure of the double-diamond *Pn3m* space group of symmetry. The lattice parameter of the DPA-plasmalogen phosphoethanolamine (C16:1p-22:5n6 PE)/MO mixed cubic phase is a_Q(Pn__3__*m*__)_ = 9.65 nm, which suggests that the plasmalogen-ethanolamine modifies the hydration level of the aqueous channel networks in the host MO lipid cubic phase.

Differences in the phospholipid headgroup type (PE, PC, or PI) and the hydrocarbon chain structure are reflected in the liquid crystalline mixed-phase formation (Figure 4). The investigated lipid/water ratio of 40/60 (wt/wt) corresponds to a hydration level, which might not ensure homogeneous hydration of the bulk self-assembled mixture of MO and DPA-phosphocholine plasmalogen (C16:1p-22:5n6 PC). The sequence of Bragg peaks, displaying a first peak centered at *q* = 0.0635 Å^–1^, evidences a distinct domain of a cubic symmetry in the mixed assembly (Figure 4, blue plot). The Bragg peaks spaced in the ratio √2:√3:√4:√6:√8:√9:√10:√11 … define a double-diamond *Pn3m* cubic phase structure with a lattice parameter a_Q(Pn__3__*m*__)_ = 14.0 nm. The obtained swollen diamond-type *Pn3m* cubic phase (D_*Large*_) appears to coexist with a domain of a less hydrated liquid crystalline structure. The Bragg peaks at *q*_1_ = 0.092 Å^–1^ and *q*_2_ = 0.184 Å^–1^, correspond to the first and the second orders of a lamellar phase with a bilayer periodicity *d* = 6.83 nm. This result indicates that the DPA-plasmalogen phosphocholine (C16:1p-22:5n6 PC)/MO assembly forms distinct mixed cubic phase domains (of the double-diamond *Pn3m* space group of symmetry), which coexist with a mixed lamellar phase. It should be noted that the structural parameters of the obtained binary plasmenyl-PC/MO lipid phases significantly differ from those of the pure MO, for which a_Q(Pn__3__*m*__)_ = 10.8 nm (at full hydration) and *d* = 4.6 nm [under limited hydration conditions, e.g., at lipid/water ratios above 60/40 (wt/wt)].

When the long-chain DPA-diacyl phosphoinositol (22:5n6-22:5n6 PI) ester lipid was mixed with the colipid MO, the bulk liquid crystalline sample entirely transformed into a lamellar structure (Figure 4, green plot). The well-resolved Braggs peaks for the mixed di-22:5n6-22:5n6 PI/MO lamellar phase (*q*_1_ = 0.079 Å^–1^ and *q*_2_ = 0.158 Å^–1^) define a bilayer periodicity *d* = 7.95 nm. This repeat spacing is essentially bigger as compared to that of single-component MO bilayers (*d* = 4.6 nm), which may be obtained at lipid/water ratios above 60/40 (wt/wt).

### Nanoparticles Loaded With Docosapentaenoyl-Plasmalogens or Docosapentaenoyl Diacyl Phosphoinositol Ester as Reservoirs for PUFA and Antioxidants

Despite that the biological activities of the novel synthetic ether or ester PUFA-phospholipids (Figure 2) are not yet known, we used these compounds to design nanoparticles as potential PUFA delivery carriers with antioxidant properties. The ongoing development of neutracetical formulations combining plasmalogen derivatives with natural antioxidants is motivated by the reports that emphasize the link between decreased ethanolamine plasmalogen concentrations and the risk of AD (Paul et al., 2019; Su et al., 2019). Recently, a marine-based plasmalogen formulation with curcumin supplement called “NeuroPlas Plasmalogen complex” (*Bio-Mer International*) has been offered to patients suffering from memory loss (brain fatigue) and having a risk of neurodegenerative diseases (e.g., AD and other types of dementia).

**FIGURE 5:**
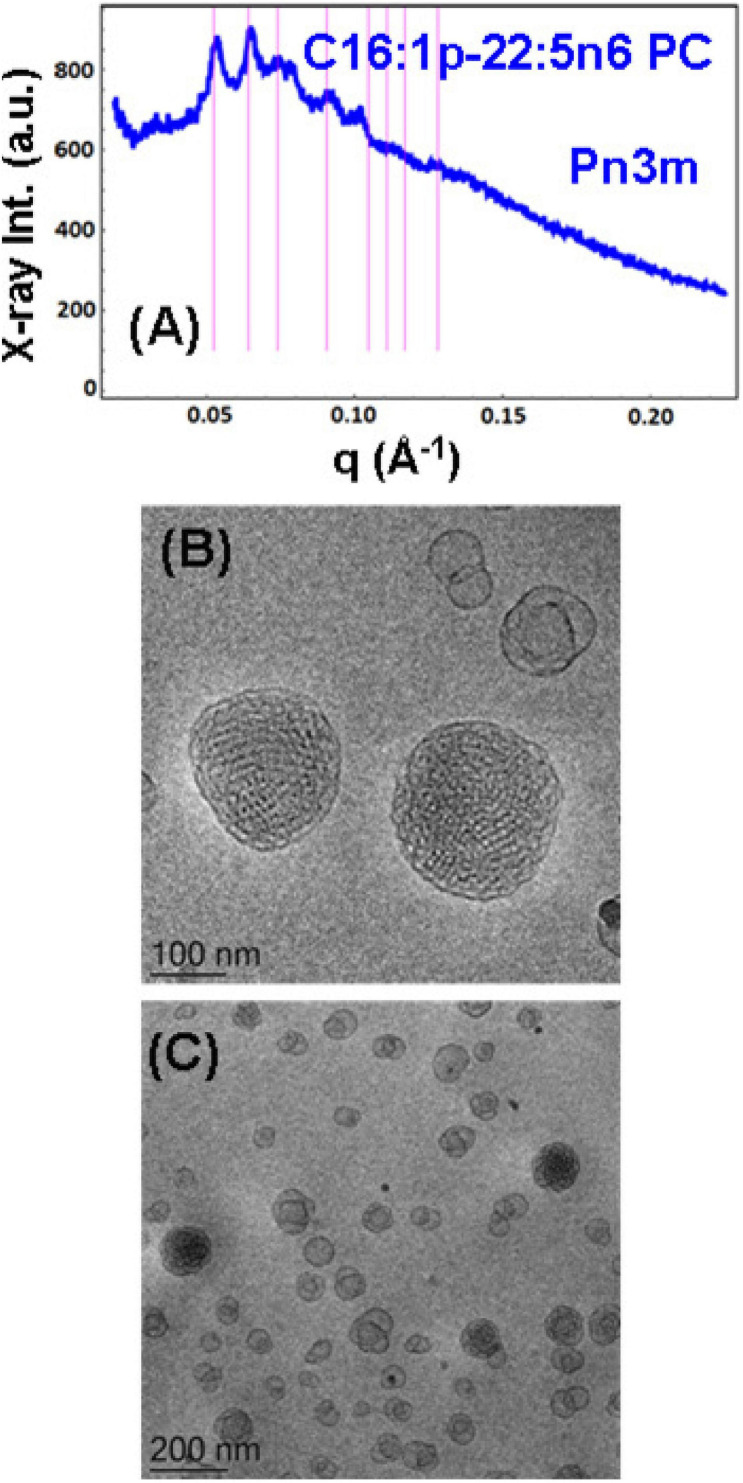
**(A)** Synchrotron small-angle X-ray scattering (SAXS) pattern and **(B,C)** cryogenic transmission electron microscopy (cryo-TEM) images of a self-assembled nanoparticulate plasmalogen-phosphocholine (C16:1p-22:5n6 PC)/monoolein (MO)/vitamin E/coenzyme Q_10_/VPGS-PEG_1000_ system with a plasmalogen-PC/MO molar ratio of 15/85 (mol/mol) and added vitamin E (10 mol.%) and coenzyme Q_10_ (1 mol.%). Aqueous phase: 1.10^− 2^ M phosphate buffer containing 2,6-di-tert-butyl-4-methylphenol (BHT). Dispersion content: 5 wt% lipid/95 wt% aqueous buffer. The set of Bragg peaks in **(A)** indexes an inner cubic structure of the double-diamond *Pn3m* cubic lattice space group. The bars indicate the (110), (111), (200), (211), (220), (221), (310), and (311) reflections, for which the peak positions are spaced in the ratio √2:√3:√4:√6:√8:√9:√10:√11 … The topologies of the cubosome particles, stabilized by VPGS-PEG_1000_ (6 mol.%), and coexisting vesicular membranes are presented in **(B,C)**.

**FIGURE 6:**
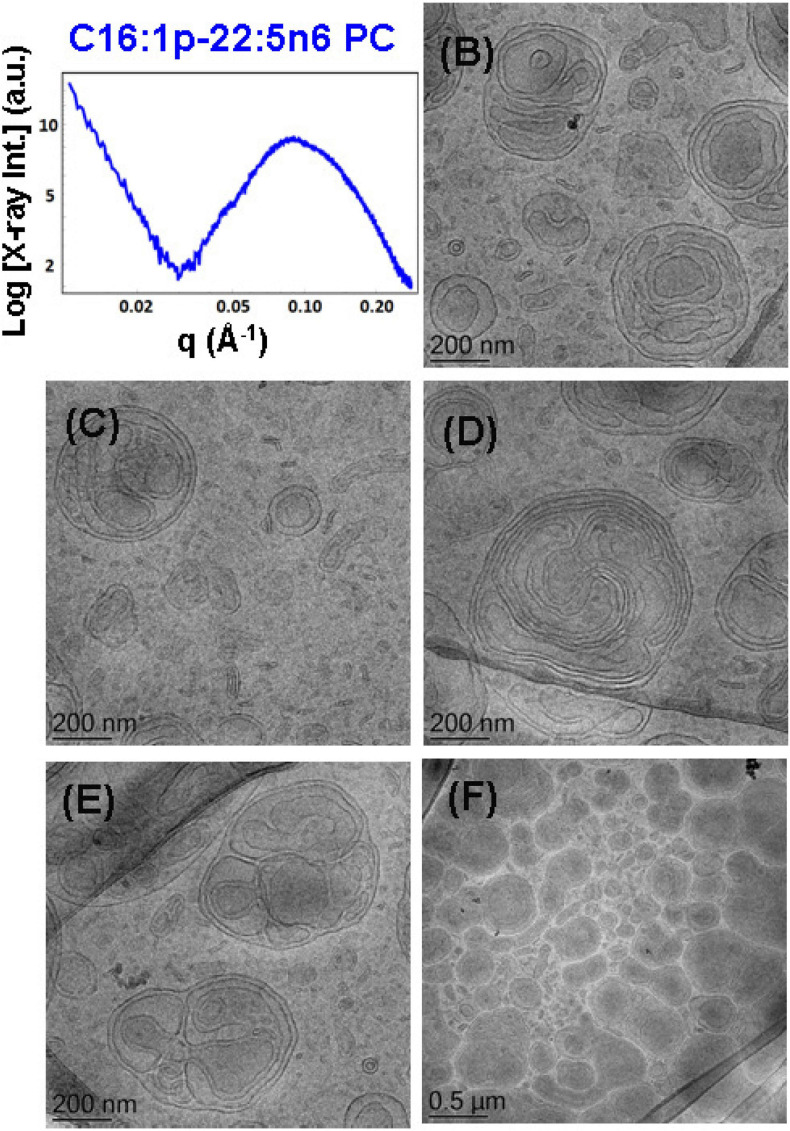
**(A)** Synchrotron small-angle X-ray scattering (SAXS) pattern and **(B–F)** cryogenic transmission electron microscopy (cryo-TEM) images of a nanoparticulate plasmalogen-phosphocholine (C16:1p-22:5n6 PC)/vitamin E/VPGS-PEG_1000_ system obtained by self-assembly and dispersion in an excess aqueous phosphate buffer (1.10^− 2^ M) phase containing 2,6-di-tert-butyl-4-methylphenol (BHT). Dispersion content: 5 wt% lipid phase/95 wt% aqueous phase.

**FIGURE 7:**
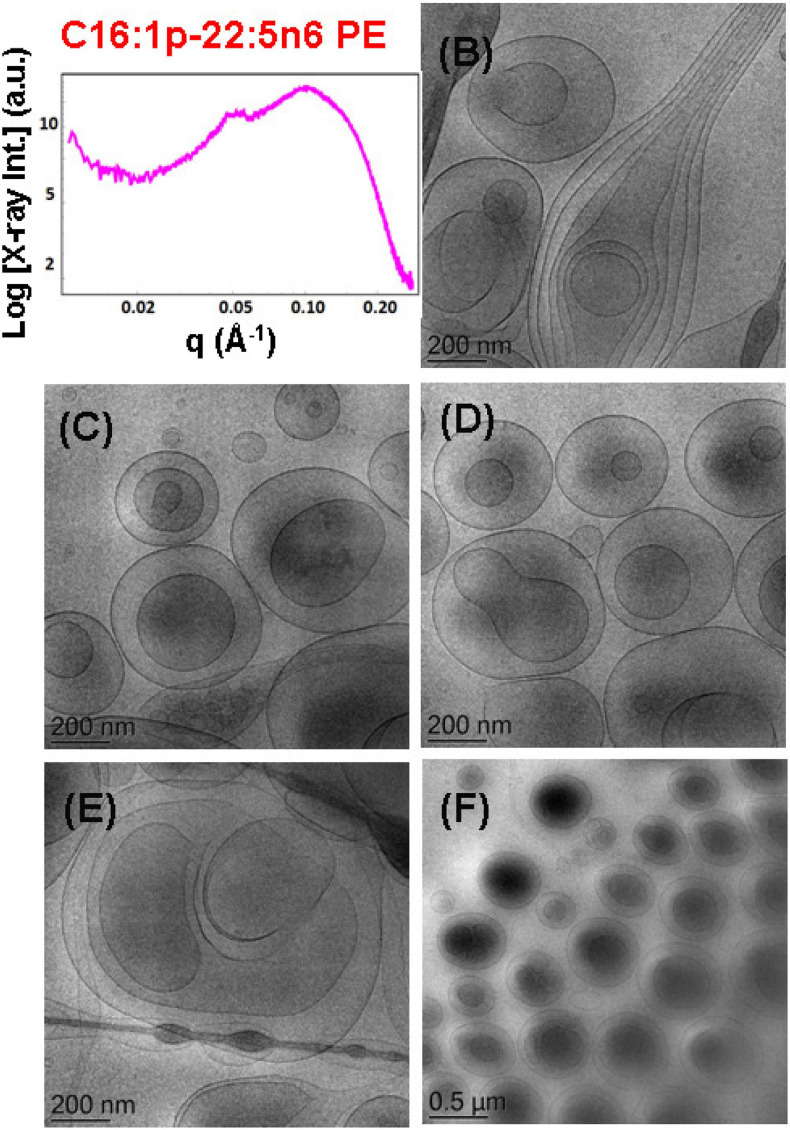
**(A)** Synchrotron small-angle X-ray scattering (SAXS) pattern and **(B–F)** cryogenic transmission electron microscopy (cryo-TEM) images of a dispersed plasmalogen-phosphoethanolamine (C16:1p-22:5n6 PE)/dioleoylphosphocholine (DOPC)/coenzyme Q_10_/VPGS-PEG_1000_ system at a docosapentaenoyl (DPA)-plasmalogen PE/monoolein (MO) molar ratio of 15/85 (mol/mol) and containing a coenzyme Q_10_ (1 mol.%) and VPGS-PEG_1000_ (6 mol.%). Aqueous phase: 1.10^− 2^ M phosphate buffer with dissolved 2,6-di-tert-butyl-4-methylphenol (BHT). Dispersion content: 5 wt% lipid phase/95 wt% aqueous buffer phase.

**FIGURE 8:**
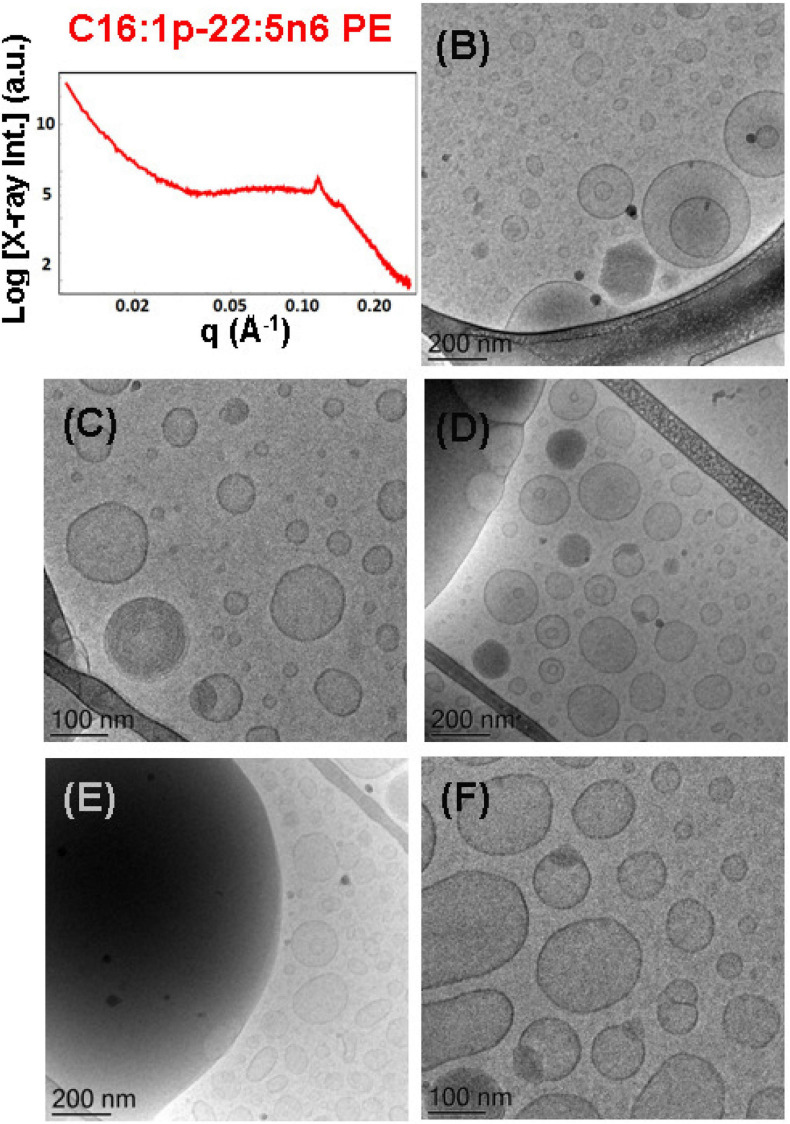
**(A)** Synchrotron small-angle X-ray scattering (SAXS) pattern and **(B–F)** cryogenic transmission electron microscopy (cryo-TEM) images of a nanoparticulate plasmalogen-phosphoethanolamine (C16:1p-22:5n6 PE)/monoolein (MO)/VPGS-PEG_1000_ system with a docosapentaenoyl (DPA)-plasmalogen PE/MO molar ratio of 20/80 (mol/mol) and dispersed by VPGS-PEG_1000_ (6 mol.%). Aqueous phase: 1.10^− 2^ M phosphate buffer with dissolved 2,6-di-tert-butyl-4-methylphenol (BHT). Dispersion content: 5 wt% lipid phase/95 wt% aqueous phase.

**FIGURE 9:**
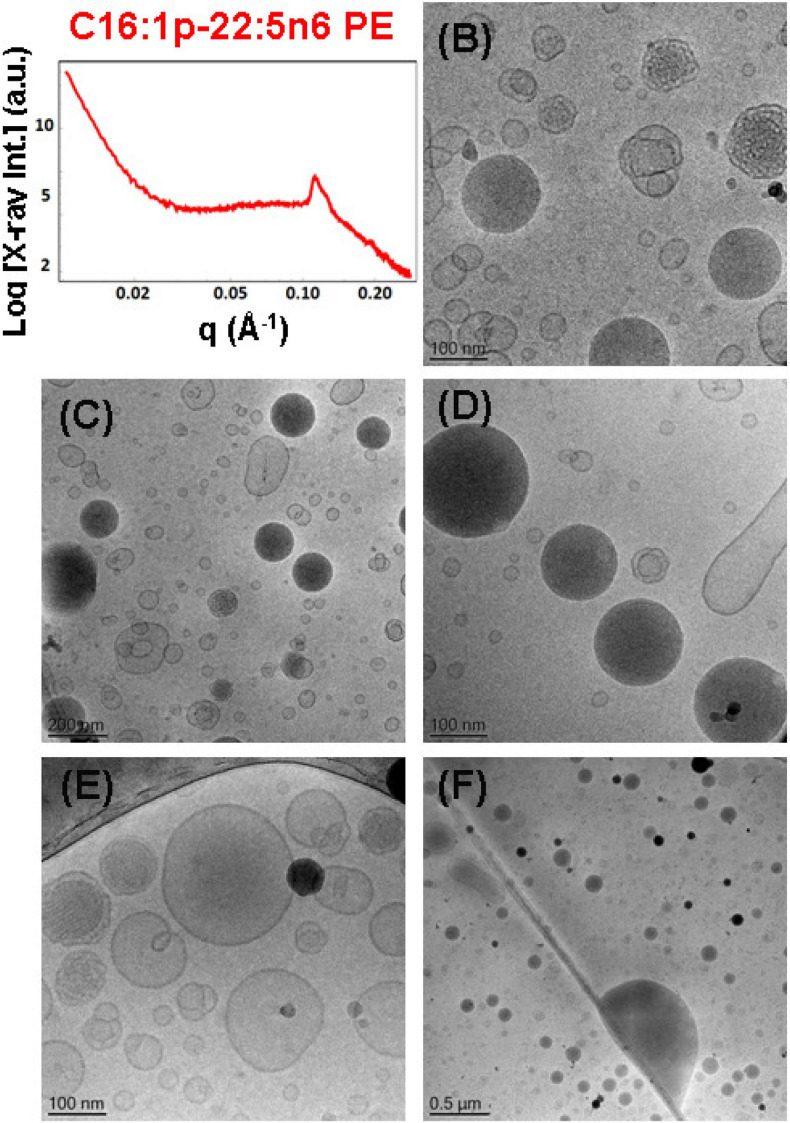
**(A)** Synchrotron small-angle X-ray scattering (SAXS) pattern and **(B–F)** cryogenic transmission electron microscopy (cryo-TEM) images of a self-assembled nanoparticulate plasmalogen-phosphoethanolamine (C16:1p-22:5n6 PE)/monoolein (MO)/vitamin E/coenzyme Q_10_/VPGS-PEG_1000_ system with a plasmalogen-PE/MO molar ratio of 20/80 (mol/mol) and added vitamin E (5 mol.%) and coenzyme Q_10_ (1 mol.%). Aqueous phase: 1.10^− 2^ M phosphate buffer containing 2,6-di-tert-butyl-4-methylphenol (BHT). Dispersion content: 5 wt% lipid phase/95 wt% aqueous phase.

**FIGURE 10:**
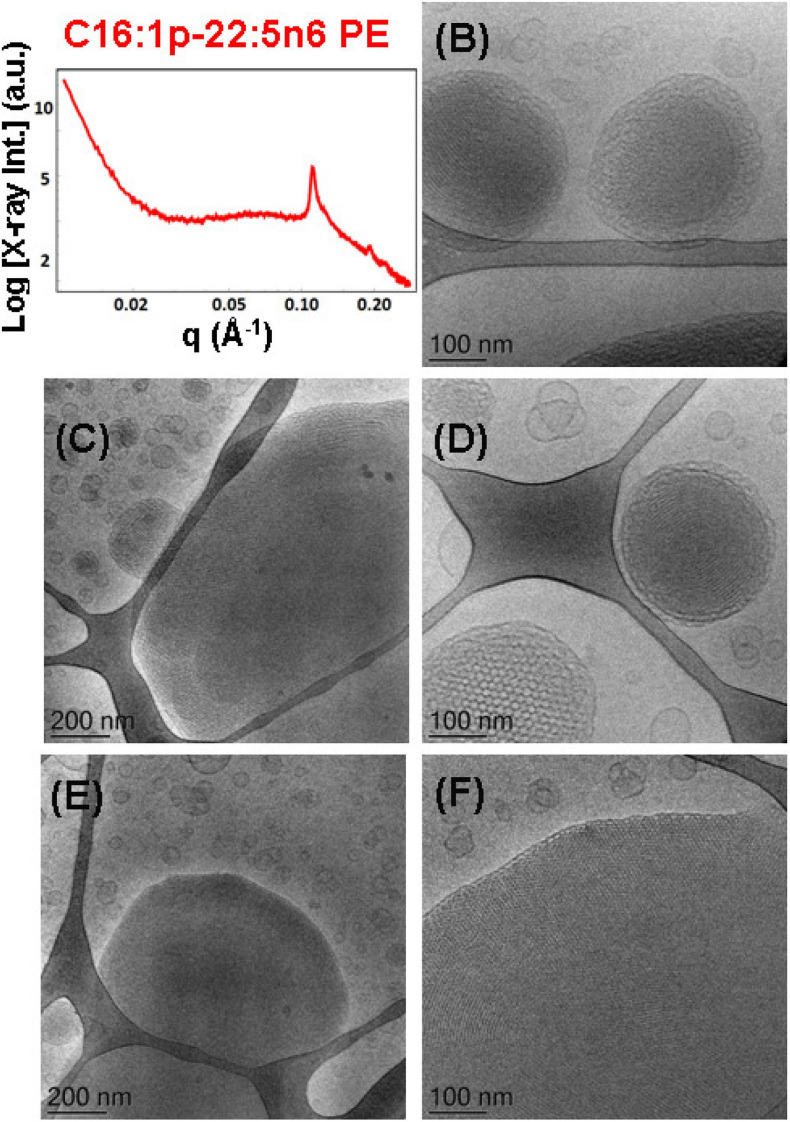
**(A)** Synchrotron small-angle X-ray scattering (SAXS) pattern and **(B–F)** cryogenic transmission electron microscopy (cryo-TEM) images of a self-assembled nanoparticulate plasmalogen-phosphoethanolamine (C16:1p-22:5n6 PE)/monoolein (MO)/vitamin E/coenzyme Q_10_/VPGS-PEG_1000_ system with a plasmalogen-PE/MO molar ratio of 15/85 (mol/mol) and added vitamin E (10 mol.%) and coenzyme Q_10_ (1 mol.%). Aqueous phase: 1.10^− 2^ M phosphate buffer containing 2,6-di-tert-butyl-4-methylphenol (BHT). The Bragg peak positions spaced in the ratio 1:√3:√4 correspond to (10), (11), and (20) reflections of an inverted hexagonal (H_II_) phase inner organization of the lipid nanoparticles (hexosomes). Dispersion content: 5 wt% lipid phase/95 wt% aqueous phase. The topologies of the hexosome nanoparticles surrounded with soft coronas and stabilized by VPGS-PEG_1000_ (6 mol.%) and fragments of a dense inverted hexagonal (H_II_) phase are presented in **(B–F)**.

**FIGURE 11:**
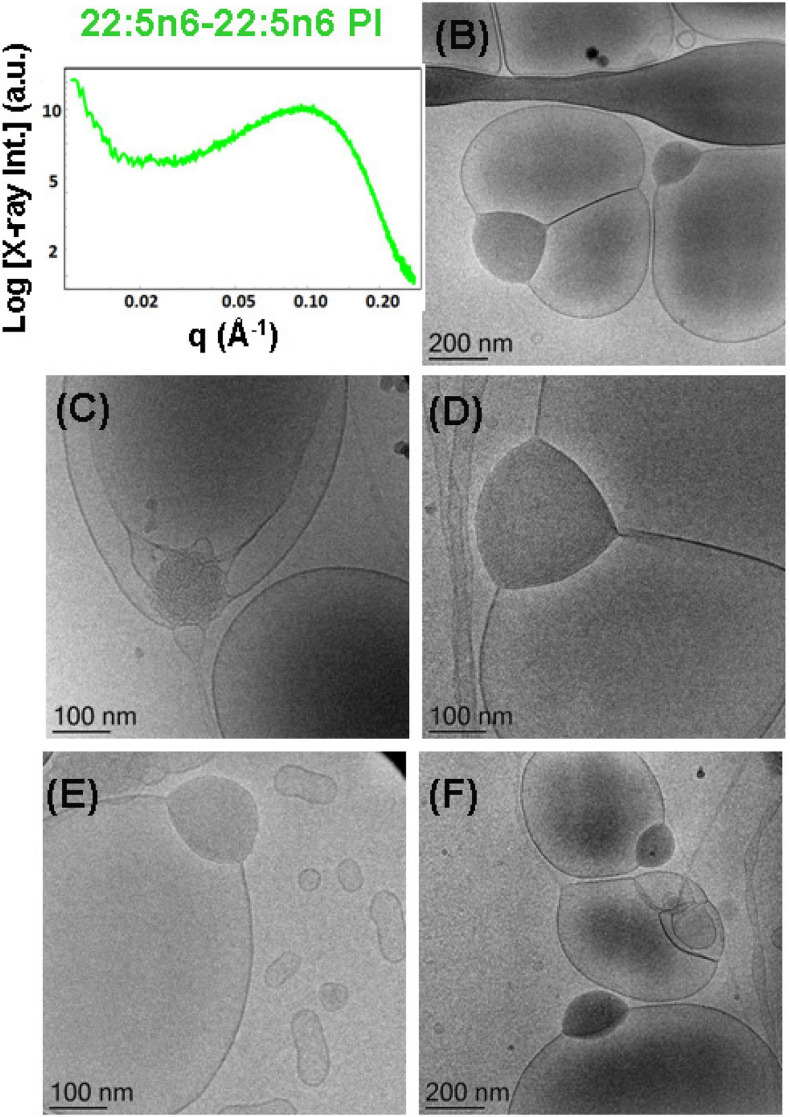
**(A)** Synchrotron small-angle X-ray scattering (SAXS) pattern and **(B–F)** cryogenic transmission electron microscopy (cryo-TEM) images of a self-assembled nanoparticulate docosapentaenoyl (DPA)-diacyl phosphoinositol (22:5n6-22:5n6 PI)/monoolein (MO)/vitamin E/coenzyme Q_10_/VPGS-PEG_1000_ system with a DPA-diacyl PI/MO molar ratio of 15/85 (mol/mol) and added vitamin E (10 mol.%) and coenzyme Q_10_ (1 mol.%). The PEGylated amphiphile VPGS-PEG_1000_ is included at 6 mol.%. Aqueous phase: 1.10^− 2^ M phosphate buffer containing 2,6-di-tert-butyl-4-methylphenol (BHT). Dispersion content: 5 wt% lipid/95 wt% aqueous buffer.

Here, stable nanoparticulate PUFA-plasmalogen (ether) and PUFA-ester-containing dispersions were fabricated, in excess aqueous medium, with the synthetic phospholipids in mixtures with co-amphiphiles [MO and D-α-tocopherol polyethylene glycol-1000 succinate (VPGS-PEG_1000_)] as well as lipophilic antioxidants (vitamin E and coenzyme Q_10_). In this way, the PUFA-phospholipids were protected by incorporation into nanocarriers, which were dispersed in a buffer environment of neutral pH. The cryo-TEM images, presented below (Figures 5–11), show the nanoparticle topologies, which were produced with selected mixed amphiphilic compositions. At full hydration, the liquid crystalline phase states were characterized by SAXS for each formulation involving DPA-phospholipids.

#### Nanoparticles Containing Plasmalogen Phosphocholine (C16:1p-22:5n6 PC)

A cubosome dispersion of the synthetic plasmalogen-phosphocholine (C16:1p-22:5n6 PC)/MO self-assembled system, involving vitamin E (10 mol.%) and coenzyme Q_10_ (1 mol.%), was achieved using the amphiphile VPGS-PEG_1000_ (6 mol.%) for fragmentation of the lipid cubic phase into nanoparticles (Figure 5). The cubosomal organization was evidenced by the SAXS pattern in Figure 5A and the cryo-TEM image in Figure 5B. The positions of the Bragg peaks spaced in the ratio √2:√3:√4:√6:√8:√9:√10:√11 … (with a first peak maximum resolved at *q* = 0.053 Å^–1^) characterize the inner structure of cubosome particles through the double-diamond *Pn3m* cubic space group. A *Pn3m* lattice parameter a_Q(Pn__3__*m*__)_ = 16.76 nm is determined for the particles with a distinct cubic symmetry (cubosomes). The Bragg peaks in the SAXS pattern in Figure 5A are superimposed on a broad maximum (*q* ∼0.092 Å^–1^) arising from the presence of double-membrane vesicles or weakly packed 3D bilayer membrane architectures, which are referred to as cubosomal intermediates. The cryo-TEM image in Figure 5C shows that nanoparticles with inner liquid crystalline structures are accompanied by vesicular membranes. The inclusion of the PEGylated agent VPGS-PEG_1000_ (6 mol.%) in the lipid system causes certain disorder of the interfaces of the small cubosomes. The presence of vesicular membranes in the surrounding of cubosomes in fully hydrated non-lamellar lipid systems is often due to the sonication process employed for nanoparticles production (Yaghmur and Glatter, 2009).

To probe the self-assembly properties of the DPA-plasmalogen-phosphocholine (C16:1p-22:5n6 PC), the synthetic lipid was dispersed in a mixture with the lipophilic antioxidant vitamin E and the PEGylated surfactant VPGS-PEG_1000_ in an excess aqueous buffer medium. A colipid monoolein was not added in this sample. The SAXS pattern in Figure 6A and the cryo-TEM images in Figures 6B–F reveal a multilamellar bilayer behavior of the dispersed plasmalogen-phosphocholine (C16:1p-22:5n6 PC)/vitamin E/VPGS-PEG_1000_ system. The broad maximum at *q* ∼0.093 Å^–1^ in the SAXS pattern is a characteristic for the presence of bilayer membrane fragments in the formulation. The absence of sharp Bragg diffraction peaks in Figure 6A suggests that the bilayers are not packed in a periodic inner lamellar structure inside the nanoparticles. The observed slope (∼*q*^–3^) in the small *q*-vector range (*q* < 0.029 Å^–1^) is characteristic for particles with rough surfaces. Actually, the cryo-TEM imaging results demonstrate an abundance of onion-type nanoparticulate topologies resulting from the dispersion of the mixed amphiphilic assemblies (Figures 6B–F). A number of fragmented multilamellar membranes appear to be of irregular shapes and non-periodic arrangements (Figures 6B,C,F). Other object shapes are found to be intermediate between small cylinders (tubules), disks, and spheres (Figures 6D,E). These features imply an inhomogeneous distribution of membrane curvature in the studied mixed assembly. The diverse topologies, comprised of double membrane vesicles, multilayer onions, and other multimembrane architectures consisting of more than two bilayers, or large vesicles encapsulating several smaller bilayer-type particles, are formed under full hydration conditions (Figures 6B–F).

#### Incorporation of Plasmalogen-Phosphoethanolamine (C16:1p-22:5n6 PE) in DOPC Membrane Nanostructures

The effect of the non-lamellar lipid DPA-plasmalogen phosphoethanolamine (C16:1p-22:5n6 PE) on the formation of mixed membrane nanostructures with the colipid 1,2-dioleoyl-sn-glycero-3-phosphocholine (DOPC) was examined by SAXS and cryo-TEM techniques at 15 mol.% DPA-plasmalogen content. The SAXS pattern of the dispersed plasmalogen-phosphoethanolamine (C16:1p-22:5n6 PE)/MO/coenzyme Q_10_/VPGS-PEG_1000_ system is shown in Figure 7A. Correlation peaks, indicating the presence of non-periodic membrane arrangements, are observed at *q*_1_ ∼0.05 Å^–1^ and *q*_2_ ∼0.099 Å^–1^. The latter maximum coincides with the position of the strongest Bragg peak for the bulk DPA-plasmalogen ethanolamine (C16:1p-22:5n6 PE) liquid crystalline phase. Nevertheless, the structural results do not demonstrate a topological transition from a lamellar to a non-lamellar phase in excess aqueous medium. The non-lamellar plasmalogen lipid does not induce a periodic cubic phase arrangement in the mixed self-assembled DPA-phosphoethanolamine (C16:1p-22:5n6 PE)/DOPC/Q_10_/VPGS-PEG_1000_ system. Thus, the lamellar phase, which is typical for the pure DOPC, represents the dominant structure in the dispersed amphiphilic system.

The cryo-TEM images in Figures 7B–F display various nanoscale object topologies with smooth interfaces (Figures 7B–D,F). Double-membrane particles and weakly packed multimembrane assemblies are mostly formed upon incorporation of the DPA-plasmalogen ethanolamine, at 15 mol.% content, in host DOPC glycerophospholipid membranes (Figures 7B–F). The topology of the lipid particles in Figure 7E suggests a locally inhomogeneous distribution of the membrane curvature. The inhomogeneous curvature distribution along the lipid bilayer scaffolds evidently results in the formation of hierarchical-type objects such as large vesicle shells encapsulating smaller particles as well as elongated tubular membranes (Figures 6E, 7B).

#### Nanoparticles Containing Plasmalogen-Phosphoethanolamine (C16:1p-22:5n6 PE)

An abundance of nanoparticles of liquid crystalline topologies was produced with DPA-plasmalogen phosphoethanolamine (C16:1p-22:5n6 PE)-involving non-lamellar (MO) lipid mixtures stabilized by the D-α-tocopherol polyethylene glycol-1000 succinate (VPGS-PEG_1000_) amphiphile (6 mol.%). Two molar ratios between the plasmenyl-PE and MO lipids were investigated, namely, 15/85 (mol/mol), and 20/80 (mol/mol).

The mixture of the synthetic DPA-plasmalogen phosphoethanolamine (C16:1p-22:5n6 PE) with MO [molar ratio, 20/80 (mol/mol)] was dispersed *via* the PEGylated amphiphile VPGS-PEG_1000_ (6 mol.%) in an excess buffer medium. The SAXS pattern in Figure 8A shows distinct Bragg peaks, which are superimposed on a broad hump (*q* ∼0.05–0.1 Å^–1^). The latter arises from the presence of fully hydrated polydispersed vesicular objects (vesicle sizes in the range of 40–200 nm). The positions of the Bragg peaks at *q*_1_ = 0.114 Å^–1^, *q*_2_ = 0.145 Å^–1^, and *q*_3_ = 0.228 Å^–1^ (a very weak third peak) determine a space group derived from the sequence 1:√3:√4 … This result implies that the obtained nanoparticles were produced by the dispersion of an inverted hexagonal (H_II_) phase, for which the inner structure is characterized by a lattice parameter a_H_ = 6.37 nm.

The cryo-TEM images in Figures 8B–F reveal the formation of dense-core particles, vesicles, and intermediate structures derived from the fragmented liquid crystalline phase of the plasmalogen-phosphoethanolamine (C16:1p-22:5n6 PE)/MO/VPGS-PEG_1000_ assembly. The increased percentage of the DPA-plasmenyl-ethanolamine (20 mol.%.) in the amphiphilic mixture might lead to a phase separation of the PUFA-PE phospholipid, which has a propensity for non-lamellar phase formation. Figure 8E shows a large fragment of a H_II_-phase domain, which coexists with vesicles produced upon the dispersion of the bulk amphiphilic mixture. A coexistence of a small hexosome nanoparticle with single-bilayer and double-membrane vesicles is shown in Figure 8B. Dense core (H_II_-phase) particles coexisting with vesicles are presented also in the cryo-TEM images in Figures 8C,D. Several mixed type nanoscale objects, involving dense H_II_-phase domain joint with a vesicle, are observed in Figures 8C,D,F. The coexisting populations of two-compartment nano-objects (dense non-lamellar-domain embedding vesicles) and vesicles of non-spherical shapes in the cryo-TEM images (Figures 8D,F) evidence that the particles are derived from a DPA-plasmalogen phosphoethanolamine (C16:1p-22:5n6 PE)-based lipid dispersion with a non-lamellar propensity.

The SAXS pattern of the self-assembled plasmalogen-phosphoethanolamine (C16:1p-22:5n6 PE)/MO/vitamin E/coenzyme Q_10_/VPGS-PEG_1000_ system with a DPA-plasmalogen PE/MO molar ratio of 20/80 (mol/mol) with additional loading of small-molecule antioxidants, such as coenzyme Q_10_ (1 mol.%) and vitamin E (5 mol.%), is presented in Figure 9A. The inclusion of vitamin E and coenzyme Q_10_ stabilizes the inner inverted hexagonal (H_II_) structural organization of the PUFA-phospholipid/MO assembly. This is evidenced by distinct Bragg peaks at *q*-vector positions of 0.113, 0.196, and 0.224 Å^–1^. The Bragg peaks are spaced in the ratio 1:√3:√4 and determine a lattice parameter of the inverted hexagonal (H_II_) phase structure a_H_ = 6.42 nm. The broad hump (*q* ∼0.03–0.09 Å^–1^) in the SAXS pattern (Figure 9A) indicates that the hexosome nanoparticles coexist with vesicular membranes in the lipid dispersion.

The morphological results for the plasmalogen-phosphoethanolamine (C16:1p-22:5n6 PE)/MO/vitamin E/coenzyme Q_10_/VPGS-PEG_1000_ assemblies in Figures 9B–F also evidence the formation of vesicular membranes, dense hexosome nanoparticles, and some non-lamellar structural intermediates. The latter involve the formation of soft corona of nanochannels, which is characteristic for weakly packed 3D membrane architectures.

The SAXS patterns in Figure 10A and the cryo-TEM images in Figures 10B–F characterize the dispersed plasmalogen-phosphoethanolamine (C16:1p-22:5n6 PE)/MO/vitamin E/coenzyme Q_10_/VPGS-PEG_1000_ particles containing coenzyme Q_10_ (1 mol.%) and vitamin E (10 mol.%) for plasmalogen-PE/MO molar ratio of 15/85 (mol/mol). The first three Bragg peaks, resolved at *q*-vectors of 0.112, 0.194, and 0.224 Å^–1^ in the SAXS pattern, correspond to the (10), (11), and (20) reflections of an inverted hexagonal (H_II_) phase structure in the lipid nanoparticles. The inner H_II_-phase structure of hexosomes is characterized by a lattice parameter a_H_ = 6.48 nm.

Multiphase nanoparticles and hierarchical structures were observed upon dispersion of the mixed amphiphilic composition [plasmenyl-PE/MO lipid molar ratio of 15/85 (mol/mol) stabilized by the PEGylated surfactant]. The cryo-TEM images of the nanoparticulate plasmalogen-phosphoethanolamine (C16:1p-22:5n6 PE)/MO/vitamin E/coenzyme Q_10_/VPGS-PEG_1000_ system are displayed in Figures 10B–F. Figures 10C,E,F show large domains with distinct inner inverted hexagonal symmetry. They represent a weakly hydrated self-assembled structure with a densely packed hexagonal lattice (a_H_ = 6.48 nm). The periphery of the hexosome nanoparticles, shown in Figures 10B,D, is less densely packed and comprises a soft corona of nanochannels. Moreover, a cubosome particle with large channels is visualized as a transitional state in the cryo-TEM image in Figure 10D. The swollen cubosomes, coexisting with vesicular particles, contribute to the scattering in the *q*-vector range ∼0.03–0.095 Å^–1^. Therefore, it can be concluded that the produced nanoparticles predominantly involve inverted hexagonal inner liquid crystalline structure surrounded by soft, less densely packed corona of nanochannels at this plasmalogen-phosphoethanolamine (C16:1p-22:5n6 PE) content. The stabilization of the formulation is provided by the included antioxidants and the coexisting vesicular membrane particles.

#### Nanoparticles Containing DPA-Diacyl Phosphoinositol (22:5n6-22:5n6 PI)

Liquid crystalline nanostructures including the synthetic PUFA-diacyl phosphoinositol ester species were obtained by dispersion of DPA-diacyl phosphoinositol (22:5n6-22:5n6 PI)/MO/vitamin E/coenzyme Q_10_/VPGS-PEG_1000_ mixed assembly using the PEGylated amphiphile VPGS-PEG_1000_. The SAXS pattern in Figure 11A displays a hump, rather than sharp Bragg peak maxima of multilamellar structures that are established with the DPA-diacyl phosphoinositol ester-containing bulk phase (Figure 4, green plot). The correlation peak at *q* ∼0.093 Å^–1^ corresponds to a correlation distance of the bilayers *L* = 6.75 nm and indicates a non-periodic bilayer membrane organization.

The cryo-TEM images in Figures 11B–F indicate phase separation of the DPA-diacyl phosphoinositol (22:5n6-22:5n6 PI) component in an amphiphilic mixture, which results in the formation of multicompartment and multiphase particles. Indeed, vesicular nanoparticles with aqueous core are joined with large oil domains and thus comprise oil droplet-embedding vesicles. These mixed objects are built up by a dense compartment (rich in PUFA-phospholipid) and a vesicular membrane, which indicates the little miscibility of the single- and double-chain lipids inside the inner structure of the nanocarriers. This leads to an abundance of nanoparticles with a mixed-type of liquid crystalline inner organizations and the presence of non-periodic membrane scaffolds.

It may be suggested that the formation of intermediate structures (e.g., oil droplet-embedding vesicles) are governed by the unsaturated chains of the PUFA-phospholipid and their miscibility with the MO matrix. Similar multicompartment topologies have been found in self-assembled systems of MO and single-chain PUFA species (Angelova et al., 2018). In the case of the dispersed eicosapentaenoic acid (EPA, C20:5)/MO mixtures, the correlation peak in the SAXS pattern has been positioned at *q*∼0.15 Å^–1^. Correspondingly, the determined correlation distance was smaller (*L* = 4.2 nm) and thus associated with thinner lipid membrane formation in the EPA/MO system with regard to the double-chain PUFA-phospholipid/MO mixtures, which are investigated in the present work.

## Discussion

Biological membranes contain significant amounts of non-lamellar-forming lipids, but their exact *in vivo* functions remain not fully understood. Lipids with PUFA chains [e.g., eicosapentaenoic (EPA, C20:5), docosahexaenoic (DHA, C22:6), and docosapentaenoic (DPA, C22:5)] acids exhibit diverse health effects and present strong current interest for structural investigations in view of biomedical applications. DPA exists in two isomeric forms, namely, C22:5n6 (*all-cis*-4,7,10,13,16-docosapentaenoic acid or 4Z,7Z,10Z,13Z,16Z-DPA) and C22:5n3 (*all-cis*-7,10,13,16,19-docosapentaenoic acid or 7Z,10Z,13Z,16Z,19Z-DPA). The use of the first isomer (DPA, C22:5n6) in the present work for the design of PUFA-plasmalogens (ether) or ester species is motivated by the increased percentage of DPA (C22:5n6) derivatives found in biological cubic membrane structures (Deng et al., 2009).

The performed SAXS investigations demonstrated the formation of non-lamellar, primitive cubic (*Im3m*), and inverted hexagonal (H_II_) phases by the hydrated DPA-ethanolamine plasmalogen (C16:1p-22:5n6 PE). Cubic liquid crystalline phases were formed also in hydrated mixed assemblies of monoolein with DPA-plasmalogen phosphoethanolamine (C16:1p-22:5n6 PE) and DPA-plasmalogen phosphocholine (C16:1p-22:5n6 PC). The colipid MO was found to maintain the stability of the mesophases embedding the polyunsaturated DPA-plasmalogen phosphoethanolamine and phosphocholine lipids and the DPA-diacyl phosphoinositol (22:5n6-22:5n6 PI). The structural effects related to the differences in the phospholipid headgroup type (PE, PC, or PI) were found to be more significant at the hydration level employed for bulk lipid phases preparation [e.g., lipid/water ratio of 40/60 (wt/wt)] as compared to the mixtures dispersed in excess aqueous environment [i.e., lipid/water ratio of 5/95 (wt/wt) allowing full lipid hydration].

The tendency for induction of curved membrane structures and short-lived intermediates appears to be important for the comprehension of the role of the plasmalogen lipids in the cellular membranes (Lohner, 1996; Almsherqi et al., 2010; Koivuniemi, 2017; West et al., 2020). By inducing transient cubic phase intermediates or higher curvature regions in the membranes of subcellular organelles, the plasmenyl lipids may exert effects on the organization and the activity of membrane proteins or of proteins anchored to the plasma membranes. It has been claimed that non-bilayer-type lipids affect peripheral and integral membrane proteins *via* changes in the lateral pressure profile (Lee, 2004). This can induce modifications in the activity of membrane proteins under oxidative stress conditions (Pohl and Jovanovic, 2019). In fact, phosphatidylethanolamine is a crucial target for reactive aldehydes. Modifications of the lipid shape and membrane properties due to lipid peroxidation-derived aldehydes can alter the membrane curvature, lipid bilayer elastic properties, and lateral pressure profile. As a consequence, this may affect the functions of membrane receptors, transporters, channels, and enzymes (Pohl and Jovanovic, 2019). The modulation of the function of transporter and ion channel proteins by non-lamellar-structure forming plasmalogen species deserves more attention in future investigations. Curvature-dependent recognition of ethanolamine phospholipids by peptide fragments represents another promising direction for future research.

The propensity for formation of an inverted hexagonal (H_II_) phase (Seddon, 1990), as well as of non-lamellar structural intermediates, has been suggested as essential for the promotion of fusion properties of the membranes, into which plasmalogen lipids are embedded (Lohner, 1996). Primarily, the H_II_-phase formation has been reported in the literature for plasmalogens with a single double bond in the hydrocarbon tails (Lohner et al., 1991).

For the DPA-plasmalogen ethanolamine, involving long PUFA chains (C16:1p-22:5n6 PE), investigated here, a coexistence of a liquid crystalline primitive cubic *Im3m* phase with an inverted hexagonal (H_II_) phase is established in the performed synchrotron SAXS study. The failure for the DPA-PE plasmalogen to form a unique stable non-lamellar mesophase could be due to the flexible conformation of its PUFA chain. The cubic-to-H_II_ phase coexistence suggests that the number of double bonds in the long polyunsaturated DPA chain (22:5n6) might correspond to the threshold limit for the formation of an inverted hexagonal (H_II_) phase structure. Indeed, H_II_ phases have been observed for plasmalogens of lower degree of unsaturation of the tails, e.g., C16:1, C18:1, or di-C18:2 (Lohner et al., 1991). The capacity of DPA-PE plasmalogen to form non-lamellar phase coexistences and structural intermediates might be of key importance for its role in the dynamics of biological membranes under stress conditions (Deng et al., 2017).

Depending on the head group type (PE, PC, and PI), the studied membrane lipid compositions were characterized by either non-lamellar or lamellar liquid crystalline phase formation in the mixed PUFA-phospholipid/MO assemblies. The incorporation of plasmalogen phosphocholine (C16:1p-22:5n6 PC) in the host lipid (MO) matrix induced a phase coexistence of a swollen double-diamond *Pn3m* cubic phase [with a D_*Large*_ lattice parameter a_Q(Pn__3__*m*__)_ = 14.0 nm] and a lamellar phase with a much thicker repeat bilayer spacing (*d* = 6.83 nm) with regard to the periodicity of pure MO phases. The topological transition from a cubic to a lamellar phase, observed for the DPA-diacyl phosphoinositol (22:5n6-22:5n6 PI)-containing mixture, can be explained by the bulky character of the PI headgroup, which lowers the critical packing parameter of this lipid and hence determines its propensity for a lamellar phase formation.

Whereas single-chain PUFAs have been found to reduce the membrane bilayer thickness (Feller et al., 2002), the incorporation of DPA-plasmenyl phospholipids resulted in an essentially increased thickness of the lipid bilayers. This was evidenced by the SAXS results for the plasmalogen C16:1p-22:5n6 PC/MO and di-1p-22:5n6:1p-22:5n6 PI/MO mixtures. The lamellar phase of the MO/DPA-diacyl phosphoinositol (22:5n6-22:5n6 PI)/MO mixture was characterized by a very large repeat bilayer spacing (*d* = 7.95 nm) as compared to the bilayer periodicity of weakly hydrated MO (*d* = 4.6 nm). Thickening of the bilayers in the liquid crystalline assemblies confirms that PUFA-plasmalogens and PUFA-esters play a role of structural determinant components in the membranes. Studies with other plasmalogen species have established thickening of 1-palmitoyl-2-oleoyl-sn-glycero-3-phosphocholine (POPC) bilayers upon incorporation of increasing amounts of plasmalogen (Rog and Koivuniemi, 2016; West et al., 2020). It should be noted that a bilayer thickness mismatch in mixed lipid membranes controls the domain size and the phase separation, which is of crucial importance for the embedded proteins. Thickening of the lipid bilayers may dramatically influence the organization of membrane proteins (Dean and Lodhi, 2018; Fontaine et al., 2020). It is known that membrane receptor clustering and domain formation may interfere with the protein function and signaling activation (Angelov and Angelova, 2017).

Self-assembly has been intensively exploited in recent years for the fabrication of liquid crystalline nanocarriers for various applications including therapeutic delivery (Yaghmur and Glatter, 2009; Fong et al., 2010, 2014, 2019; Azmi et al., 2015; Angelova et al., 2018, 2019a; Shao et al., 2018; Wang et al., 2018; Li et al., 2019; Talaikis et al., 2019; Zhai et al., 2019). Drug loading and release capacities have been shown to depend on the internal structural organization of the delivery vehicles (Yaghmur and Glatter, 2009; Rakotoarisoa et al., 2019; Zhai et al., 2019). Multiphase structures have been observed in lipid mixtures of multiple amphiphilic components as well as upon loading of therapeutic proteins and peptides in nanostructured assemblies (Angelov et al., 2014; Angelova et al., 2019b).

Stable synthetic liquid crystalline structures with new topological properties were obtained by self-assembly of the DPA-phospholipids with colipids and amphiphiles. The results indicated that abundant liquid crystalline structures and shapes can be fabricated as a consequence of the lyotropic lipid polymorphism of the DPA-based plasmalogens and ester phospholipids (plasmalogen-phosphoethanolamine C16:1p-22:5n6 PE, plasmalogen phosphocholine C16:1p-22:5n6 PC, and DPA-diacyl phosphoinositol 22:5n6-22:5n6 PI; Figures 5–11).

**FIGURE 12:**
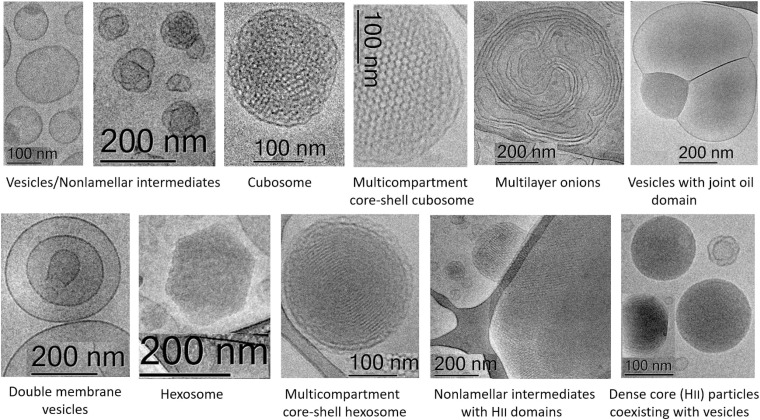
Summary of the different nanoscale topology types of docosapentaenoyl (DPA) plasmalogen- and ester-based liquid crystalline multiphase structures generated by spontaneous assembly in excess aqueous medium. Examples of individual cubosome and hexosome particles are given together with multiphase nanoparticles and intermediate structures formed as a result of inhomogeneous curvature distribution in the studied polycomponent lipid mixtures.

Figure 12 and Table 1 outline the nanoscale object types obtained as a result of the structural polymorphism of the investigated lyotropic lipid/DPA-phospholipid-containing systems at room temperature. Apart from the antioxidant properties of the encapsulated plasmalogen lipids, the biomedical usefulness of the resulting topologies will depend on the envisioned strategy for plasmalogen administration at target therapeutic sites. Some crystalline arrangements may be more desirable than others in controlled release applications. It has been indicated that cubosomes and hexosomes display different drug release profiles (Fong et al., 2014; Azmi et al., 2015; Wang et al., 2018), the release from hexosome nanocarriers being essentially slower. The inner structural organization of the cubosome carriers is advantageous for enhanced entrapment and protective encapsulation of hydrophilic proteins, peptides, and nucleic acids (Angelova et al., 2011). The shape of the nanoparticles is crucial for the transport and diffusion properties of the drug delivery carriers. Generally, drug transport via particles with elongated tubular shapes is different from that from sphere-shaped objects. Hexosomes with elongated, cylinder-like topologies may be more efficient in controlled release applications because of their increased circulation time as well as increased residence time at biological membrane barriers (Li et al., 2019). For combination therapy applications with bioactive lipids, core–shell multiphase cubosomes and hexosomes as well as multicompartment vesicles with joint oil domains (i.e., oil droplet embedding vesicles) have advantages for dual and multidrug delivery (Angelova and Angelov, 2017; Angelov et al., 2017; Angelova et al., 2018).

**TABLE 1:** Summary of the nanoscale liquid crystalline structures identified for the different mixed lipid compositions upon spontaneous assembly in excess aqueous medium.

Multicomponent amphiphilic mixture	Liquid crystalline structures	Figures
Plasmalogen-phosphocholine (C16:1p-22:5n6 PC, 15 mol%)/MO/vitamin E/coenzyme Q10/VPGS-PEG_1000_	Cubosomes coexisting with vesicles; Cubosomal intermediates	Figure 5B Figure 5C
Plasmalogen-phosphocholine (C16:1p-22:5n6 PC)/vitamin E/VPGS-PEG_1000_	Multilayer onions; Multimembrane fragments; Double membrane vesicles	Figures 6B–F Figure 6C
Plasmalogen-phosphoethanolamine (C16:1p-22:5n6 PE, 15 mol.%)/DOPC/coenzyme Q_10_/VPGS-PEG_1000_	Multimembrane structures; Double membrane vesicles	Figure 7B Figures 7C,D
Plasmalogen-phosphoethanolamine (C16:1p-22:5n6 PE, 20 mol.%)/MO/VPGS-PEG_1000_	Hexosome coexisting with vesicles; Dense H_II_-phase domain joint with vesicle; dense core (H_II_) particles coexisting with vesicles	Figure 8B Figures 8C,D,F Figures 8C–F
Plasmalogen-phosphoethanolamine (C16:1p-22:5n6 PE, 20 mol.%)/MO/vitamin E/coenzyme Q_10_/VPGS-PEG_1000_	Non-lamellar intermediates; Dense core (H_II_) particles coexisting with vesicles	Figures 9B,E Figures 9C,D,F
Plasmalogen-phosphoethanolamine (C16:1p-22:5n6 PE, 15 mol.%)/MO/vitamin E/coenzyme Q_10_/VPGS-PEG_1000_ system	Multicompartment core-shell hexosomes; Non-lamellar intermediates with H_II_ domains; H_II_-phase domain fragments	Figure 10B Figures 10C–F Figures 10E,F
DPA-diacyl phosphoinositol (22:5n6-22:5n6 PI, 15 mol.%)/MO/vitamin E/coenzyme Q_10_/VPGS-PEG_1000_	Multicompartment vesicles with joint oil domains	Figures 11B–F

The fact that plasmalogens may behave as stimuli-responsive phospholipid biomaterials has been exploited for elaboration of controlled release systems (Anderson and Thompson, 1992; Thompson et al., 1996). In perspective, it would be of interest to investigate the stimuli-responsive properties of the prepared nanoformulations. Multiple features of the designed antioxidant-involving nanoparticles with multiphase organization may be advantageous for the achievement of stimuli-activated drug release and pH-sensitive or temperature-controlled release properties in novel biomedical applications.

## Conclusion

The performed structural investigations established that DPA plasmenyl phospholipids dramatically modulate the membrane curvature upon spontaneous assembly with colipids. They show a propensity for domain formation in membranes. For same DPA content of the hydrocarbon tails, the lipid miscibility in the two-component membrane assemblies varies depending on the phospholipid type (ether or ester) and the nature of the headgroups (PC, PE, or PI). The revealed multiphase liquid crystalline structure formation may be crucial for certain membrane-governed cell processes in the heart, lung, skeletal muscles, retina, immune cells, and brain (especially in the plasmalogen-rich human white matter frontal cortex and the human gray matter parietal cortex). The induction of non-lamellar or lamellar domains, enriched in PUFA-plasmalogens or ester phospholipids, provides a better understanding about the effects of these phospholipid species on different events occurring at the cellular and subcellular organelle level, e.g., molecular trafficking, signaling, receptor clustering and activation in membranes, lipid metabolism (cholesterol efflux), and biogenesis of cubic membranes.

The presented cryo-TEM data demonstrate the formation of multiphase nanoparticles and nanostructures with distinct symmetries or hierarchical organizations that result from variable curvature in the multicomponent membranes. The variety of inner liquid crystalline multicompartment topologies and nanoparticle shapes (cubosomes, hexosomes, double-membrane vesicles, multilayer onions, mixed objects with jointed vesicular and oil compartments, and core–shell structures) open the door to exploit plasmalogen-based soft nanoparticle architectures for novel bioinspired applications.

## Materials and Methods

### Lipids, Chemicals, and Sample Preparation

Plasmalogen derivatives with DPA chains and two types of headgroups (PE and PC) as well as a double-chain polyunsaturated DPA-phosphoinositol ether analog (Figure 2) were obtained by custom synthesis from Avanti Polar Lipids Inc. (Alabama), namely, 1-O-1′-(Z)-hexadecenyl-2-(4Z,7Z,10Z,13Z,16Z-docosapentaenoyl)-sn-glycero-3-phosphocholine, C16:1p-22:5n6 PC (M.W. 791.58), 1-O-1′-(Z)-hexadecenyl-2-(4Z,7Z,10Z,13Z,16Z-docosapentaenoyl)-sn-glycero-3-phosphoethanolamine, C16:1p-22:5n6 PE, (M.W. 749.54), and 1,2-bis(4Z,7Z,10Z,13Z,16Z-docosapentaenoyl)-sn-glycero-3-phosphoinositol (ammonium salt), di-22:5n6 PI, (M.W. 958.56). The synthetic glycerophospholipids were of high purity (>99%) and were characterized by the provider via thin-layer chromatography (TLC), mass spectrometry (MS), and NMR analyses. According to the provider, the achieved purity of the synthetic plasmalogen was the highest possible for performance of structural SAXS investigations. The products were received as lipid solutions in chloroform. Monoolein (1-oleoyl-rac-glycerol, MO, C18:1c9, MW 356.54, powder, ≥99%), DOPC, Vitamin E, coenzyme Q10, and D-α-tocopherol polyethylene glycol-1000 succinate (Vitamin E-TPGS or VPGS-PEG_1000_) were purchased from Sigma-Aldrich. For sample preparation, we took into account that the alkenyl (vinyl ether) bond is susceptible to hydrolysis under strong acidic conditions. A buffer medium of a neutral pH was prepared using the inorganic salts NaH_2_PO_4_ and Na_2_HPO_4_ (p.a. grade) and 2,6-di-tert-butyl-4-methylphenol (BHT; Sigma-Aldrich). The MilliQ water (Millipore Co.), serving for the preparation of the aqueous phase, was purged by nitrogen gas in order to eliminate the dissolved oxygen. The obtained aqueous medium, containing the antioxidant BHT, ensured the oxidative stability of the formulations.

Bulk phase samples from the DPA-phospholipid/MO mixtures were prepared by self-assembly at a lipid/water ratio of 50/50 (wt/wt) using aqueous phosphate buffer (NaH_2_PO_4_/Na_2_HPO_4_, 1.10^–2^ M, pH 7) with dissolved small amount of BHT. The chloroform solutions of the lipid compounds were handled under stream of nitrogen gas to prevent oxidation of the vinyl ether bond of plasmalogen. Lipid nanoparticles were prepared at a lipid/water ratio of 95/5 (wt/wt). Both the bulk mesophases and the dispersed lipid particles were produced by the method of hydration of a dry lipid film followed by physical agitation (Angelova et al., 2018). The lipids were mixed at chosen proportions [95/5, 90/10, 85/15, and 80/20 (mol/mol)], and the solvent was evaporated under flux of nitrogen gas. The obtained fine and homogeneous lipid films were lyophilized overnight under cooling. Vortexing and agitation in an ice bath were applied in repeating cycles of 1 min each. Ultrasonic cycles with a total duration of <10 min (Branson 2510 ultrasonic bath, “set sonics” mode, power 60 W) were sufficient to produce dispersions of lipid nanoparticles in the presence of PEGylated amphiphile VPGS-PEG_1000_.

### Synchrotron Small-Angle X-Ray Scattering

Bulk liquid crystalline phases formed by hydrated lipid assemblies and dispersed nanoparticles were investigated at the SWING beamline of synchrotron SOLEIL (Saint Aubin, France) as recently reported (Rakotoarisoa et al., 2019). The samples were placed in capillaries or in a designed gel holder with (X, Y, and Z) positioning. Temperature was 22°C. The sample-to-detector distance was 3 m, and the X-ray beam spot size on the samples was 25 × 375 μm^2^. The patterns were recorded with a two-dimensional Eiger X 4M detector (Dectris, Baden-Daettwil Switzerland) at 12 keV allowing measurements in the *q*-range from 0.00426 to 0.37 Å^–1^. The *q*-vector was defined as *q* = (4π/λ) sin θ, where *2*θ is the scattering angle. The synchrotron radiation wavelength was λ = 1.033 Å, and the exposure time was 250 or 500 ms. The *q*-range calibration was done using a standard sample of silver behenate (*d* = 58.38 Å). An average of five spectra per sample was acquired. Data processing of the recorded 2D images was performed by the FOXTROT software (David and Pérez, 2009).

Toward nanostructure determination, the positions of the resolved Bragg diffraction peaks were fitted according to the relationships of the Miller indexes. A sequence of Bragg peak positions spaced in the ratio √2:√3:√4:√6:√8:√9:√10:√11:√12:√14 is a characteristic of a bicontinuous *Pn3m* double-diamond cubic lattice. The primitive *Im3m* cubic lattice is characterized by peaks spaced as √2:√4:√6:√8:√10:√12:√14:√16:√18:√20:√22, whereas the sequence of Bragg peaks positions spaced in the ratio √6:√8:√14:√16:√20:√22:√24:√26 defines a gyroid *Ia3d* cubic lattice structure. The Bragg peak positions of multilamellar bilayer structures are spaced in the ratio 1:2:3:4:… For an inverted hexagonal (H_II_) phase, the corresponding relationship is 1:√3:√4….

### Cryogenic Transmission Electron Microscopy

The methodology of the cryo-TEM study was analogous to the previously described work (Angelova et al., 2019a). In brief, a sample droplet of 2 μl was put on a lacey carbon-film-covered copper grid (Science Services, Munich, Germany), which was hydrophilized by glow discharge (Solarus, Gatan, Munich, Germany) for 30 s. Most of the liquid was then removed with blotting paper, leaving a thin film stretched over the lace holes. The specimen was instantly shock frozen by rapid immersion into liquid ethane and cooled to approximately 90 K by liquid nitrogen in a temperature- and humidity-controlled freezing unit (Leica EMGP, Wetzlar, Germany). The temperature and humidity were monitored and kept constant in the chamber during all sample preparation steps. The specimen was inserted into a cryo-transfer holder (CT3500, Gatan, Munich, Germany) and transferred to a Zeiss EM922 Omega energy-filtered TEM (EFTEM) instrument (Carl Zeiss Microscopy, Jena, Germany). Examinations were carried out at temperatures around 90 K. The TEM instrument was operated at an acceleration voltage of 200 kV. Zero-loss-filtered images (DE = 0 eV) were taken under reduced dose conditions (100–1,000 e/nm^2^). The images were recorded digitally by a bottom-mounted charge-coupled device (CCD) camera system (Ultra Scan 1000, Gatan, Munich, Germany) and combined and processed with a digital imaging processing system (Digital Micrograph GMS 1.9, Gatan, Munich, Germany). The sizes of the investigated nanoparticles were in the range or below the film thickness, and no deformations were observed. The images were taken very close to focus or slightly under the focus (some nanometers) due to the contrast-enhancing capabilities of the in-column filter of the employed Zeiss EM922 Omega. In EFTEMs, the deep underfocused images can be totally avoided.

## Data Availability Statement

The raw data supporting the conclusions of this article will be made available by the authors, without undue reservation.

## Author Contributions

AA: conceptualization of manuscript. YD: lipids conception and management. AA, BA, and MD: experimental investigation. TB and MD: instrument management and software. BA, MD, and AA: analysis and data processing. BA, YG, and YD: funding and materials. AA: writing—original draft. BA, YD, and AA: writing—review and editing. AA: supervision. All authors agreed to be accountable for the content of the work.

## Conflict of Interest

The authors declare that the research was conducted in the absence of any commercial or financial relationships that could be construed as a potential conflict of interest. The handling editor declared a past co-authorship with several of the authors.
